# Synthesis and Characterization of a Nano-Polyplex system of GNRs-PDMAEA-pDNA: An Inert Self-Catalyzed Degradable Carrier for Facile Gene Delivery

**DOI:** 10.1038/s41598-018-26260-4

**Published:** 2018-05-25

**Authors:** Ali Dinari, Tahereh Tohidi Moghadam, Mahdi Abdollahi, Majid Sadeghizadeh

**Affiliations:** 1 Department of Nanobiotechnology, Faculty of Biological Sciences, Tarbiat Modares University, Tehran, Iran; 2 Department of Polymer ReactionEngineering, Faculty of Chemical Engineering, Tarbiat Modares University, Tehran, Iran; 3 Department of Genetics, Faculty of Biological Sciences, Tarbiat Modares University, Tehran, Iran

## Abstract

Engineering molecules at nano-scale is a promising approach in targeting and curing diseases. In this research, fabricated new hybrid system called nano-polyplex represents an example of the molecular engineering at nano-scale. Polymer of PDMAEAs with four different molecular weights were synthesized using the RAFT method, attached onto the gold nano-rod surface, which modified and produced a safe novel system with an average size less than 100 nm. The hybrid system was characterized by ultra violet-visible spectrophotometer (UV-Vis), dynamic light scattering (DLS), ^1^H NMR, gel permeation chromatography (GPC), Fourier transform-infrared (FT-IR) spectroscopy, Zeta potential analyzer and transmission electron microscopy (TEM). Features of higher transfection and lower toxicity compared to the previously reported polyplex of PDMAEA, as well as the gold standard PEI, have been shown in all molecular weights and defined N/P ratios (10–200). The ideal physicochemical properties for escaping from the cell barriers, covering the large volume of genetic material (pDNA) and high efficiency of loading polyplexes on GNRs’ surface make it an ideal carrier. The results of this effort pave way in designing a new generation of nanoparticle-based delivery systems for nucleic acid therapy and gene editing.

## Introduction

Biotechnology has introduced molecular cloning methods to alter the genetic information of cells and living organisms in the context of gene therapy, for treatment of both acquired and inherited diseases such as Alzheimer and cancer. Nucleic acid-based therapy has emerged as a promising treatment strategy in repairing or substituting defective genes, disrupting gene expressions and gene editing in its mature form^1^. Nevertheless, there are some problems that limit the use of such types of treatments. First, the efficiency of nucleic acid therapeutic agents is remarkably reduced by electrostatic repulsion within the cell membrane. Furthermore, the presence of nuclease enzymes in cellular environments destroys these molecules before they reach the target area^2^. Hence, the design of an ideal carrier is required to properly protect, transport and release the cargo at the target site. So far, viral and non-viral vectors have been used to transfer foreign DNA to cells of interest. Viral vectors are known to present the best transfection efficiency. However, due to the lack of safety, i.e., toxicity and immunogenicity, their medical application is limited. On the other hand, non-viral vectors have shown advantages of low immune response, unrestricted gene size, ease of production and functionalization, etc. The simplest method of the non-viral gene transfer system uses naked DNA. However, the main problem of the transfected gene associated with this method is its exposure to degradation by nucleases and minimum cellular uptake^3^. Liposome vesicles are another non-viral vector with simple and facile formulation as well as ease of functionalization. Nevertheless, it presents some disadvantages which include reproducibility, cell shrinkage, colloidal instability, low transfection efficiency and relative toxicity^4^. Cationic polymers with amine groups in their backbone can easily be folded with DNA phosphate groups and assembled to form polyplexes which protects DNA during the transfection procedure. In fact, condensing DNA into nanoparticles can be considered as a promising feature of cationic polymers^5^. There are a variety of natural (e.g., dextran, gelatin and chitosan) and synthetic (e.g. L-lysine (PLL), polyethylenimine (PEI), poly (2-N,N-dimethylaminoethyl methacrylate) (PDMAEMA)) polymers employed as DNA carriers. Among these polymers, chitosan and polyethylenimine (PEI) are most often used in gene therapy research, exhibiting better gene delivery responses than their counterparts^6,7^. Recently, an acrylic acid-based polymer called poly dimethylaminoethyl acrylate (PDMAEA) has attracted significant attention as an excellent candidate for gene delivery purposes. The key features of this polymer include: I) sustenance of ionic strength for a specific period of time for efficient transfection of cells, II) binding with nucleic acids (DNA & siRNA) through ionic interactions and forming complexes, III) degrading to nontoxic building blocks/byproducts, IV) gradual change in ionic state (from cationic to anionic state), and V) degrading in aqueous environments autonomously, without responding to cellular stimuli (e.g., pH, redox potential, or presence of enzymes)^8,9^.

Considering the current problems and concerns of traditional gene delivery systems, nano-biotechnology has paved the way to solve many challenges in versatile biomedical applications. Today, building up functional agents for theranostic systems is based on nanoparticles with unique properties. Tiny particles of gold with sets of shapes and sizes have introduced many outstanding features in a variety of biomedical and bio-sensing arenas. So far, synthesis of different gold nanoparticles such as spheres, cages, wires, prisms and rods has been reported. Among these, gold nanoparticles of rod morphology with unique optical properties have been remarkably noted in theranostic applications. Facile synthesis and high surface-area-to-volume ratio, as well as ease of functionalization with various biomolecules and polymers are some of the key features of gold nanorods (GNRs) that makes them promising candidates for drug/gene carrier systems^10^. In this regard, this effort focuses on fabrication of a biocompatible, hybrid nanocarrier system containing GNRs and PDMAEA, with the aim of achieving maximum therapeutic efficiency in gene delivery with minimal adverse effects. PDMAEA was synthesized using the reversible addition fragmentation chain transfer (RAFT) method and functionalized with GNRs. The binding, protection, transfection, release profile, cytotoxicity, and stability of hybrid complexes were studied using valid assays. To the best of our knowledge, transfection of the full length of pDNA by PDMAEA-GNRs has not been previously reported.

## Results and Discussion

### Characterization of GNRs

**Figure 1:**
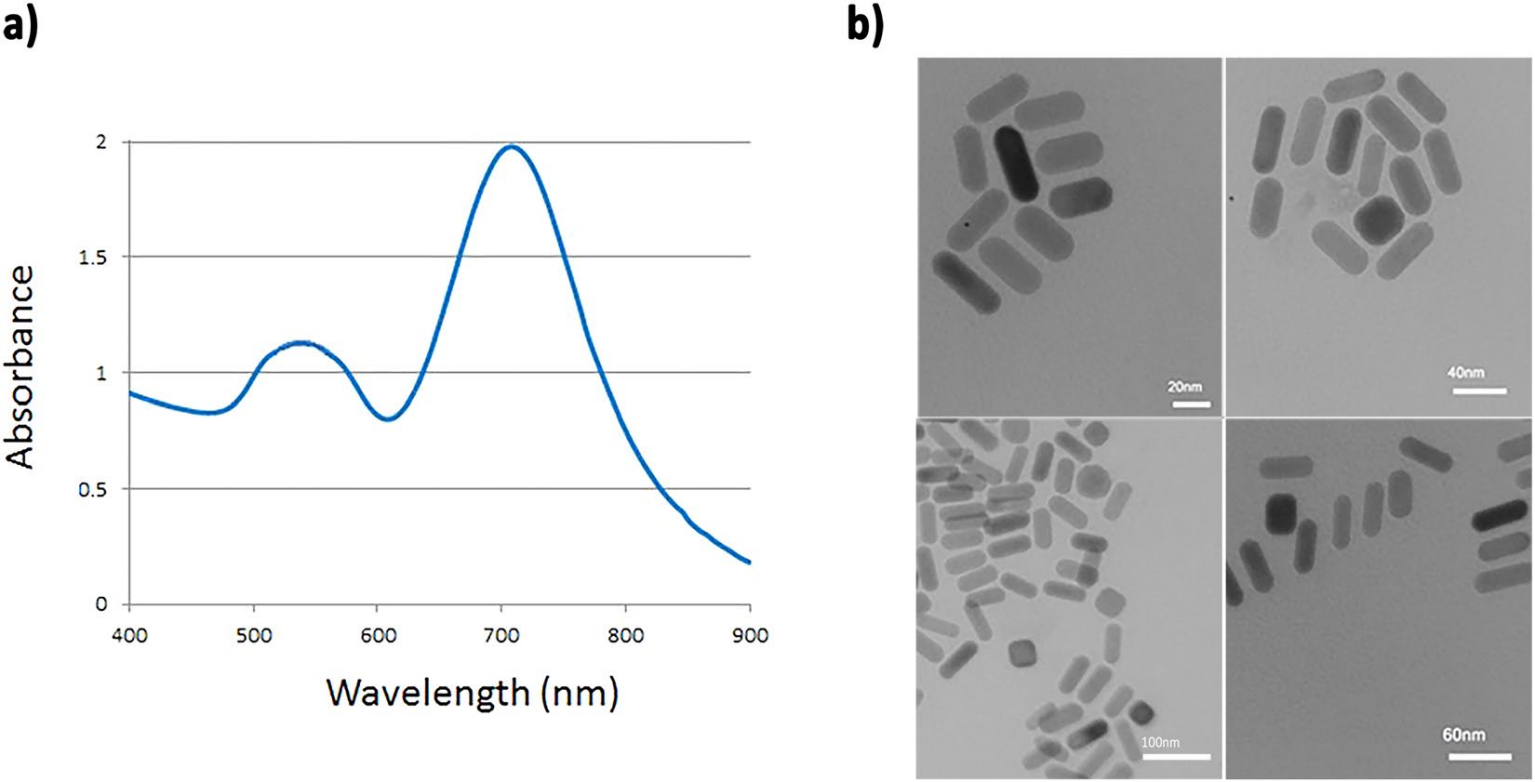
Absorption spectra of GNRs, (**b**) TEM micrograph of GNRs.

Surface plasmon resonance bands of gold nanorods were monitored in the visible and near infrared region, representing oscillation of the conduction band electrons along the short and long axis of GNRs (Fig. 1a). Appearance of a strong longitudinal surface plasmon resonance band (LSPR) at 720 nm, along with a transverse SPR band of weaker intensity at 520 nm is characteristic of formation of nanostructure with rod morphology. Figure 1b depicts the transmission electron microscopy image of the nanostructures. The mean aspect ratio of GNRs was estimated to be 3.2 ± 0.6, with average dimensions of 38.38 nm in length and 12.00 nm in width.

### Characterization of PDMAEA

RAFT polymerization is known to offer many advantages for biomedical applications. For instance, controlled molecular weight and length as well as polydispersity, ability to design a wide range of compounds with a variety of reactive end-groups for modification and functionalization are considered to be some benefits of this method. According to the vast majority of studies, versatility of RAFT agents can be proof of the superiority of this technique^11,12^. In this study, PDMAEA was synthesized with four different molecular weights using the RAFT polymerization technique, as shown in Table 1. For the sake of convenience, polymers with different molecular weights have been marked as I, II, III and IV for all data representations. Analysis of the polymers by ^1^H NMR and GPC showed controlled behavior of RAFT polymerization (Table 1).

**Table 1:** ^1^H NMR and GPC data for RAFT Polymerization of PDMAEA with four different molecular weights.

Polymer number	^1^H NMR		GPC		RAFT reation [1000]:[10]:[1] Monomer: Raft agent: AIBN			Temperature	Time	Solvent	Antisolvent
	Repeating unit	Mn (g/mol)	PDI	Mn (g/mol)	Monomer	Raft agent	AIBN				
I	23.75	3401	1.18	3922	14.1252 ml	352.26 mg	30.552 mg	60 °C	2 h	Dioxane	n-hexan
II	36.62	5244	1.39	4208	7.0626 ml	176.13 mg	15.276 mg	60 °C	5 h	Dioxane	n-hexan
III	49.15	7038	1.16	5612	3.5313 ml	88.065 mg	3.81 mg	60 °C	6 h	Dioxane	n-hexan
IV	76.29	10924	1.22	8324	3.5313 ml	88.065 mg	3.81 mg	60 °C	7 h	Dioxane	n-hexan

### Functionalization of GNRs with PDMAEA

Previous studies have shown that the presence of an excess amount of cationic surfactant provides notable positive surface charge for GNRs, which in turn can result in nonspecific cell uptake and protein adsorption upon interaction with cells, disruption of their membranes and eventually cell death^13^. This is an important issue that limits the utilization of GNRs for biocompatible medical purposes. Therefore, displacement of CTAB with a more biocompatible molecule is necessary for further fruitful biomedical applications. So far, a number of strategies have been proposed to reduce the toxic effect of CTAB molecules, which include modification of GNRs with different ligands or polymers such as PEG, PSS, etc.^14^.

**Figure 2:**
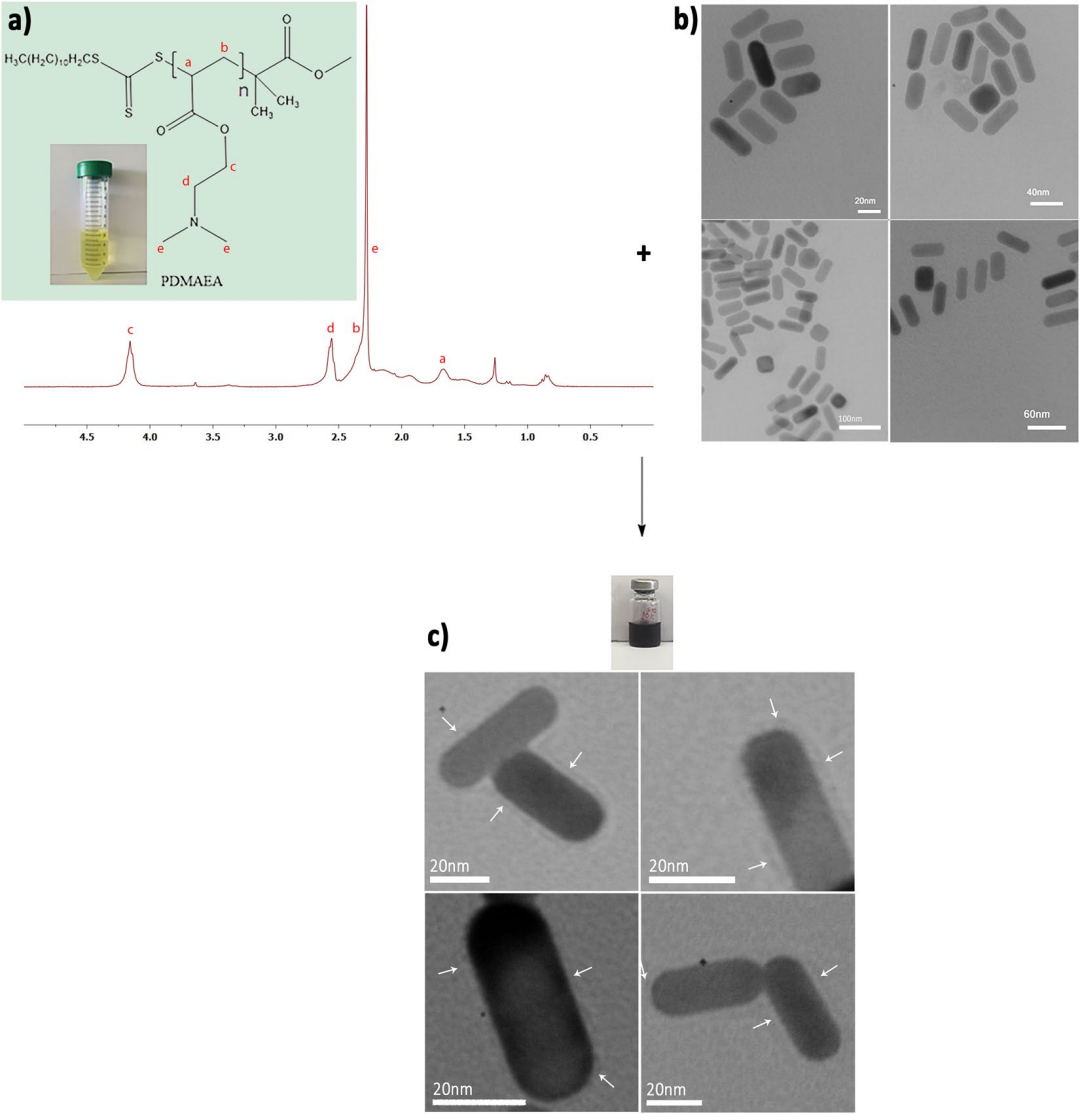
Summary of the processes involved in the synthesis of PDMAEA (**a**), and GNRs (**b**) and also attaching PDMAEA onto GNRs surface (**c**). ^1^H NMR spectrum, and TEM images represents evidences for accuracy of above processes.

In order to modify the surface of GNRs with polymer, PDMAEA was attached to the GNRs based on a modified protocol^15,16^. Figure 2 displays a summary of the synthesis reactions of PDMAEA (a), GNR (b) and attaching of PDMAEA to GNR surface (c).

**Figure 3:**
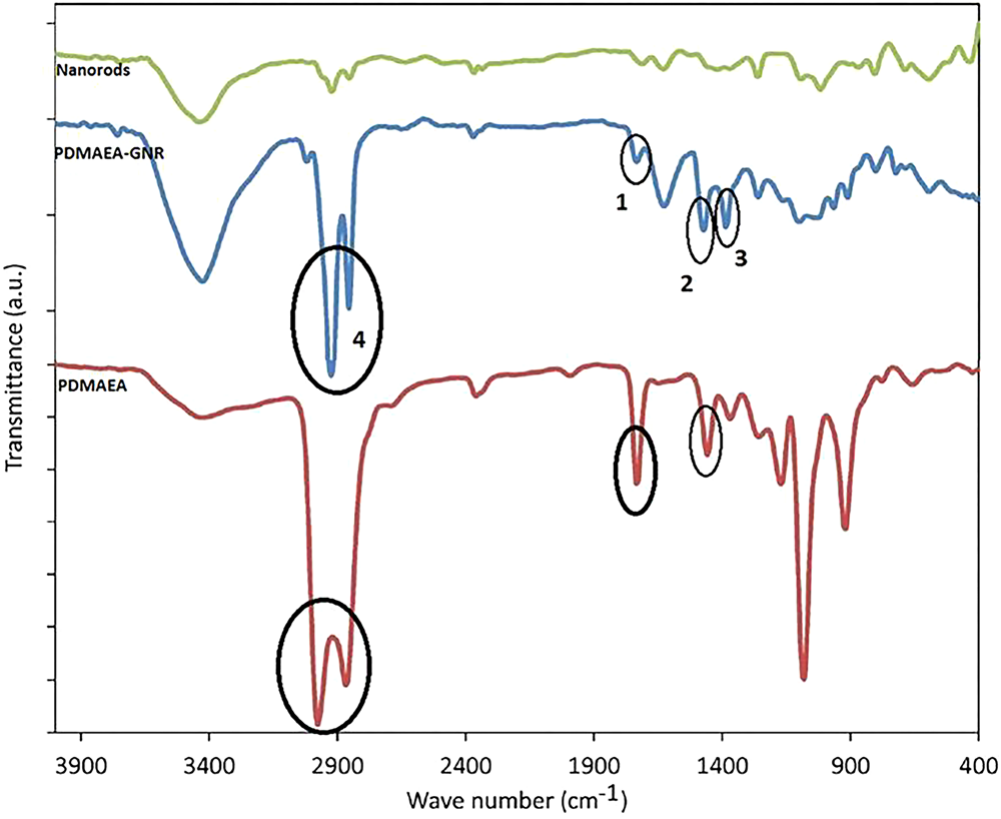
The above figure depicts FT-IR spectra of bare GNRs and PDMAEA as well as their complex forms. The characteristic peaks in the GNRs-PDMAEA complex appeared at the same position of corresponding peaks observed for bare PDMAEA. Comparison of the three FT-IR spectrum confirmed conjugation of the polymer to the matrix of nanostructures.

Typically, in this study, a “grafting to” approach was employed for functionalization of the nanostructures with PDMAEA, based on the high affinity of gold atoms for sulfur^16^. As a result, gold can form stable bonds with sulfur compounds such as thiols, dithioesters and trithiocarbonates. Figure 3 depicts the FT-IR spectra of bare GNRs and PDMAEA as well as their complex forms. The characteristic peaks in the GNRs-PDMAEA complex appeared as C=O (1734.00 cm^−1^), stretching bonding of C-S (1473.72 cm^−1^), and C-H bonding (2850–2950 cm^−1^) at the same position of the corresponding peaks observed for bare PDMAEA. The C-H bonding refers to C-H of monomers in the backbone of the polymer. Resonance binding of Au-S (1386.61 cm^−1^) indicated valuable evidence for the process of binding. Hence, the comparison of the FT-IR spectrum of the bare PDMAEA with its complex form with GNRs confirmed conjugation of the polymer to the matrix of nanostructures.

Zeta potential is another useful technique for monitoring PDMAEA interaction with GNRs. According to the DLS results shown in Table 2, there was notable decrease in the zeta potential value for all the *nano-polyplexes* (GNR-PDMAEA-pDNA) compared to that of the untreated GNRs (without PDMAEA), which had a much higher value due to the presence of cationic surfactant (CTAB), i.e. +37.8 mV. Such changes in the zeta potential values can represent interaction of the GNRs with PDMAEA and formation of the nano-polyplexes.

**Table 2:** DLS results of polyplexes, nano-polyplexes and PEI as control.

	N/P ratio	Polymer I		Polymer II		Polymer III		Polymer IV	
		Size (nm)	Zeta (mV)	Size (nm)	Zeta (mV)	Size (nm)	Zeta (mV)	Size (nm)	Zeta (mV)
Polyplexes of DNA-PDMAEA	10	215.94 (0.17)	6.7	378.7 (0.61)	7.7	178.20 (0.38)	9.8	158.22 (0.51)	21.1
	50	274.74 (0.86)	3.11	269.1 (0.71)	17.3	300.39 (0.63)	16.3	276.72 (0.55)	5.1
	100	436.9 (0.22)	1.79	475.3 (0.22)	3.3	880.3 (0.34)	22.8	539.3 (0.25)	13.9
	200	597.81 (0.48)	4.5	527.5 (0.33)	11.3	306.30 (0.83)	5.14	513.2 (0.35)	6.98
Nano-polyplexes of DNA-PDMAEA-GNRs	10	87.66 (0.43)	5.36	116.11 (0.57)	2.51	77.970 (0.41)	0.96	85.06 (1.00)	−9
	50	33.06 (0.45)	3.78	198.08 (0.72)	0.59	83.68 (0.47)	6.64	89.32 (0.47)	3.3
	100	98.15 (0.50)	2.62	99.98 (0.62)	5.2	112.44 (0.52)	5.32	95.22 (0.60)	1.43
	200	112.10 (0.38)	1.41	87.28 (0.60)	−2.37	53.13 (0.36)	5.95	204.40 (0.55)	4.03
**polymer of PEI**									
	**N/P ratio**			**Size (nm)**			**Zeta (mV)**		
Polyplexes of DNA-PEI	10			85.53 (0.38)			31.2		
	50			101.43 (0.35)			34.9		
	100			120.77 (0.56)			39.4		
	200			97.69 (0.41)			34.2		

In addition to the mentioned analytical techniques, TEM image which is shown in Fig. 2(c) approved the attachments of PDMAEA on GNRs surfaces. The arrows in the image clearly define the attachment process.

### DNA-binding and Protection Assay of PDMAEA

**Figure 4:**
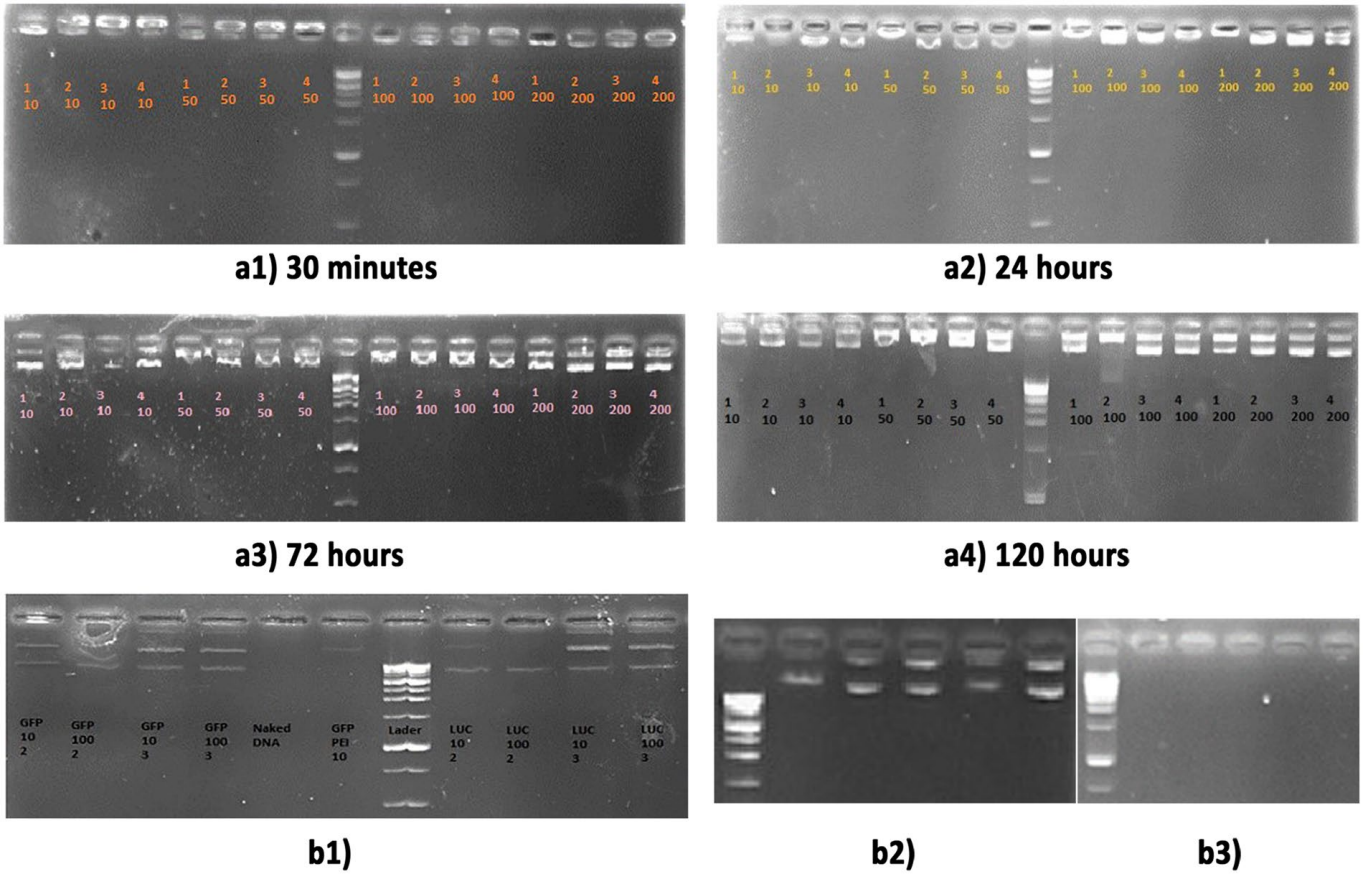
DNA-binding and Protection Assay of PDMAEA. (**a1**–**a4**) Assays of DNA-binding potency of PDMAEA during the following period of time: 30 min, 24, 72 and 120 hours. **b1**–**b3**) Protection assays of PDMAEA. Comparison of PDMAEA protective role with PEI as standard agent in gene transfer (**b1**). DNA protection and dissociation pattern after one week of polyplex formation, before and after treated with DNase I respectively (**b2** and **b3**).

The prominent feature of an ideal gene delivery carrier system is the capability to transport the foreign gene to its target without any damage. In this regard, capability is defined as the ability of the carrier to escape the processes that affect the natural state of exogeneous DNA^7^. Covering and condensing the DNA molecule with polymers is considered as a useful protection strategy against nuclease digestion. Recently, PDMAEA is found to have shown excellent properties as well as degradation in an independent manner in the presence of external or environmental triggers. The self-catalyzed hydrolysis mechanism of this polymer in a defined time interval is useful in the delivery of genes and drugs. To study the role of PDMAEA in condensing pDNA molecules and also measurement of the size of polyplexe, pDNA (GFP and luciferase separately) was mixed with polymers of four molecular weights (as shown in Table 1) at different N/P ratios (10 to 200 as mentioned). Figure 4 depicts the capability of PDMAEA for DNA binding/condensing by agarose gel retardation assay. The gel images have been obtained with time intervals of 30 min, 24, 72 and 120 hours, respectively, after formation of the polyplexes. From Fig. 4, it can be seen that the migration of the polyplexes in the agarose gel is retarded. This indicates the strong binding of pDNA with four different molecular weights with all N/P ratios (10 to 200). As shown in Fig. 4(a1–a4), the binding capability is gradually reduced until completion of pDNA release. Based on the running pattern in the gel, it is presumable that increasing the molecular weight and N/P ratios leads to formation of durable complexes that provides a better coverage as well as a better release of pDNA.

Previous reports on the effect of molecular weights and charge ratios of another polymer, i.e., chitosan, have shown similar results^17^. A study on PEI as a popular cationic gene carrier system has provided valuable information about the role of molecular weights and N/P ratios on polyplex formation, durability and dissociation (release of DNA from polymer)^18^. The main release mechanism for DNA molecules is based on a self-catalyzed degradation pattern of polymer in water, leading to the formation of the negatively charged poly acrylic acid and N, N-dimethylamino ethyl ethanol. The average size of the nano-polyplex system (pDNA-PDMAEA-GNR) and its distribution was measured using the dynamic light scattering technique (see Table 2). According to DLS data, the size of the nano-polyplex was estimated to be 100 nm without a noticeable difference at different N/P ratios. This could be due to the presence of GNRs which act as matrix-making dense polyplex structures (Fig. 1a). However, larger complexes were also observed, independent of molecular weight and N/P ratio. It seems that the larger size of such complexes resulted from the misconnection of polyplexes on GNRs surface and assemblies of the polyplexes by themselves. Comparison of the bare polyplexes (PDMAEA- pDNA) with nano-polyplexes revealed that the latter has a smaller size (Table 2). It should be noted that sizes of all samples at an N/P ratio of 10 and 50 were smaller, especially at the higher molecular weights. The remarkable difference between the average size of nano-polyplexes and polyplexes implies the positive impact of GNRs in condensing the overall nano-polyplex system.

Assessment of the potential of PDMAEA to protect pDNA was investigated and compared with the PEI (25 kDa-branch) as gene transfers agents. In this regard, experiments showed an excellent potential of PDMAEA for protecting pDNA from nuclease digestion (Fig. 4b). Treatment of polyplexes (PDMAEA-pDNA) with DNase I was carried out in tow separate parts as following: (1) the first part comprised the samples treated with DNase I immediately after half an hour of complex formation. The obtained results showed an excellent protection of polymer for covering pDNA samples as well as an enhanced parallel protective effect upon increase in the molecular weight (Fig. 4(b1)). (2) The second part comprised the samples stored at room temperature for one week and subsequently treated with DNase I. The results showed no pDNA band upon exposure to DNase I, and full digestion of pDNA occurred reflecting the gradual changing nature of the PDMAEA (Fig. 4(b2 and b3)). Different molecular weights of PDMAEA with N/P ratios of 10 and 100 were evaluated. The protective effect was found to increase with increase in the molecular weight of the polymer. Furthermore, in all the molecular weights, there was no perceptible difference between N/P ratios of 10 and 100. On the other hand, comparison of PDMAEA with PEI (N/P ratios of 5, 10) showed that the PDMAEA ability for gene protection was higher than that of PEI. All obtained results were similar to the results of previous researchers^9^.

Based on previous studies, packaging and protecting of nucleic acids (DNA, siRNA) and the stability of their complexes are the main parameters to protect them against cellular compartments such as endosomal/lysosomal and nuclease degradation^19,20^. Cationic polymers such as PEI, chitosan and PDMAEMA are good candidates for masking the anionic charges of nucleic acids. Complexes generated by these polymers have an appropriate protection against degradation^21^. The point that should not be forgotten is that the strength of complexes and balance of electrostatic interactions between polymer and DNA are important issues that affect the release pattern of DNA inside the cells^22^. However, defective release of DNA has common problems associated with the above-mentioned polymers. Meanwhile, strong electrostatic interactions (which are used to make durable polyplexes), conversion of the ionic composition, and full dissociation and release of DNA inside the cell without limitation, are superior features of PDMAEA.

### MTT-assay

**Figure 5:**
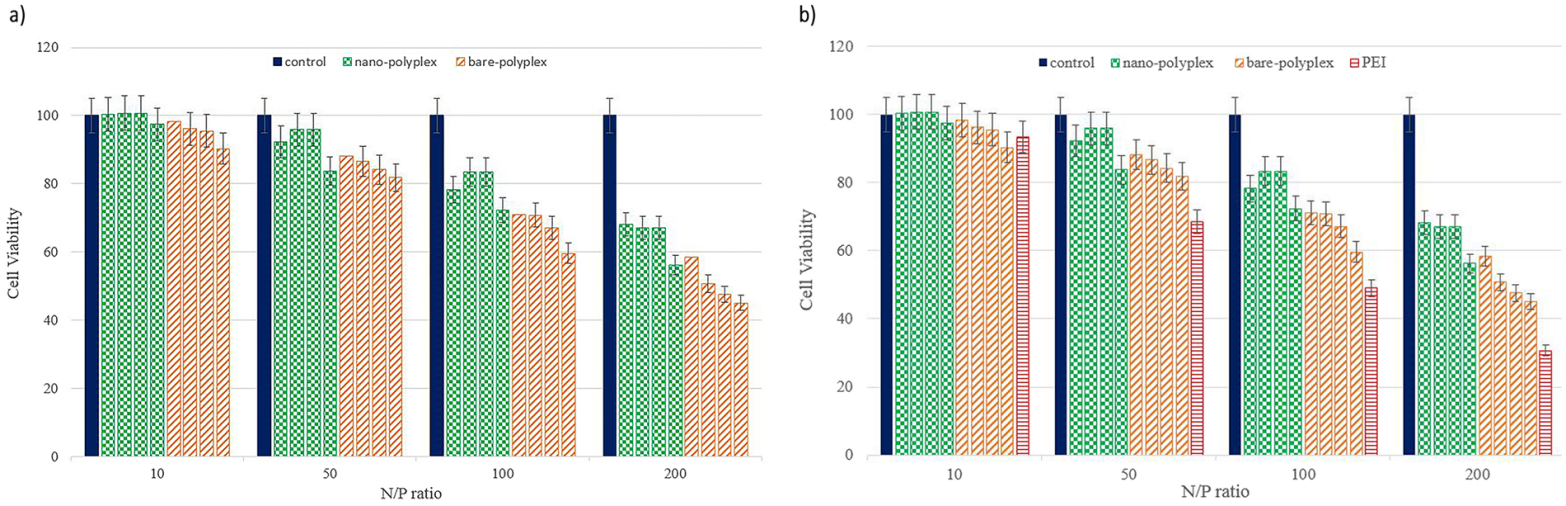
Pattern of cytotoxicity effects of four molecular weight of PDMAEA in bare-polyplex and nano-polyplex systems and comparision with PEI. Different colors indicates control (), nano-polyplex (), bare-polyplex () and PEI (). According to figure, experiments data divided in three (**a**) and/or four (**b**) groups and arranged based on N/P ratios. (**a**) Each group contains control (no treatment), nano-polyplex and bare-polyplex and also note that in each groups, molecular weight increased from left to right. As shown in figures (**a**,**b**), toxicity effect of N/P ratio is more prominent than the molecular weight. (**b**) Cytotoxicity effects of fabricated systems have been compared to polyplexes of PEI-pDNA as positive control and indicated that nano-polyplex system affected the cells in a safer fashion.

Conversion of tetrazolium salt to formazan in MTT assay represents mitochondria activity and cell survival in abnormal conditions such as drugs treatments. Therefore, this assay is useful in investigating the safety or toxicity of pharmaceutical agents^23^. In addition, cytotoxicity of vector-mediated gene transfer can be evaluated by MTT assay. The toxic effect of a cationic gene carrier refers to their positive charge, and influences their capability and efficiency in gene therapy attempts^4^. Previous research on cytotoxicity of PDMAEA has shown that polymers with a molecular weight of 5600 or lower do not induce toxic effect. The cell cytotoxicity increases with increase in the N/P ratio as well as the molecular weight of the polymer^9^. To monitor the safety of the nano-polyplex system, all four different molecular weights (I, II, III, and IV) with the specified N/P ratios were treated with the HEK293 cell line. As shown in Fig. 5, toxicity and cellular damage increased with increase in the molecular weight and N/P ratio. Comparison of the nano-polyplex system (pDNA-PDMAEA-GNRs) with ordinary complexes (PDMAEA-pDNA) and PEI- pDNA (the control) indicated that the nano-polyplex system affected the cells in a safer fashion. The bar chart clearly shows that the role of the N/P ratio is more prominent than the molecular weight. This is consistent with a previous study by Truong *et al*., who reported a negligible effect of PDMAEA on cytotoxicity in all molecular weights and N/P ratios, except that of the N/P ratio of 200^9,24^. Furthermore, a lower toxic effect of PDMAEA exists with respect to PEI. This occurs due to the fact that the amine groups of the polymer chain in PEI act as a positive charge source, thus producing a high density of charge and cell membrane disruption^25^.

### Transfection Assays

**Figure 6:**
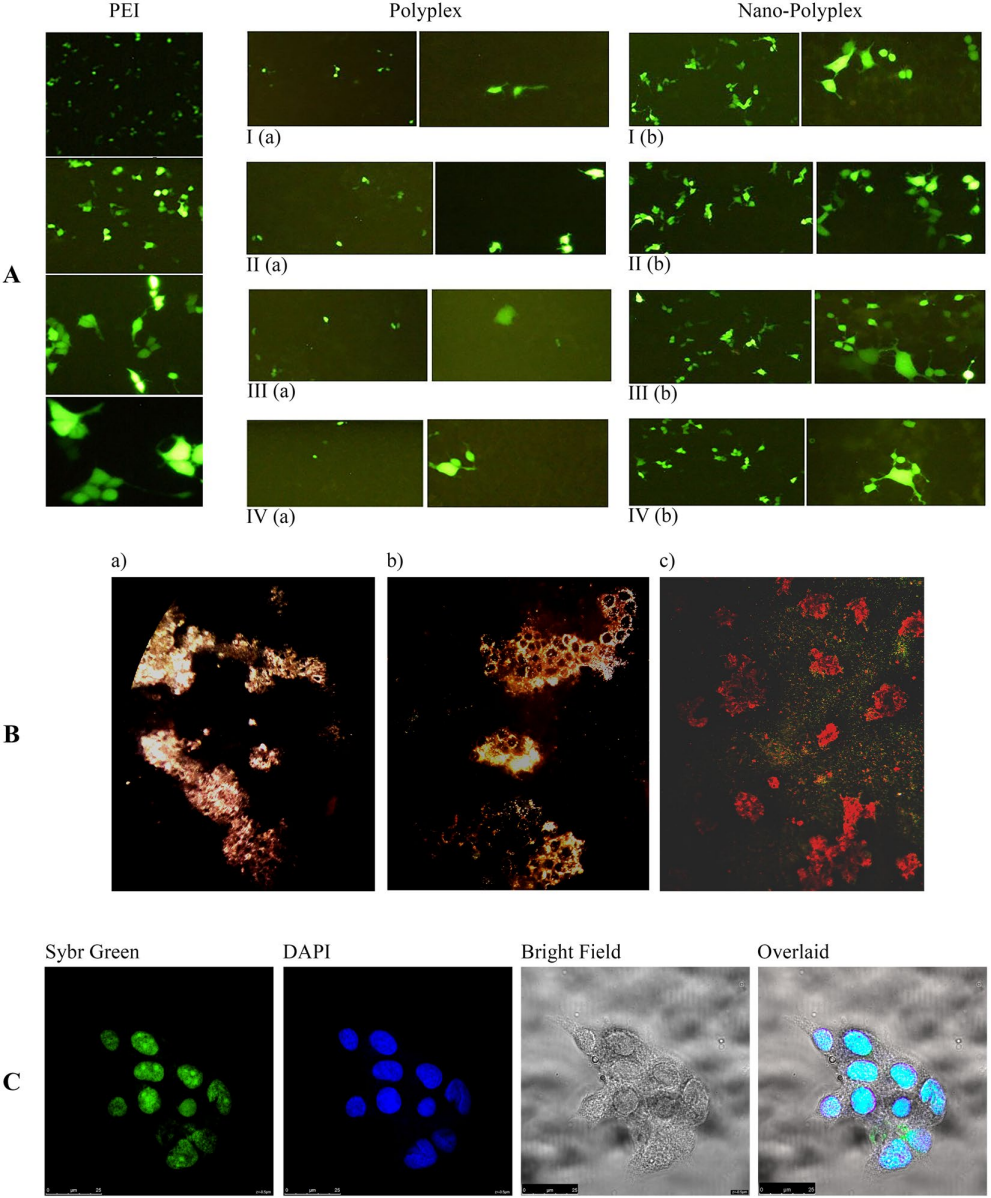
Comparison of transfection of pDNA-PEI (N/P ratio of 10) with bare-polyplexes I (a), II (a), III (a), and IV (a) and nano-polyplexes I (b), II (b), III (b), and IV (b), at N/P ratio of 50 into HEK-293 cells. All images were taken with 4, 10, 20 and 40X magnification. (**B**) Dark field microscopy images of cells treated with nano-polyplex, (**C**) Confocal microscopy images of cells treated with fluorescent-labeled nano-polyplex.

Herein, PDMAEA has been used as a carrier to transport pDNAs (GFP and luciferase) into the nuclei of the KEK-293 cell line in order to investigate its potential in transportation of pDNA, consistent with previous research which reported transport of siRNA molecules^24^. Green Fluorescent Protein (GFP) was mixed with GNRs-PDMAEA, bare PDMAEA and PEI as a control, for HEK-293 cells transfection. Experiments were conducted according to the modified protocols of previous studies^26^. Figure 6 depicts the transfected cells imaged by fluorescence microscopy. The specified N/P ratios and four molecular weights were studied for both nano-polyplexes of GNRs-PDMAEA-pDNA and polyplexes of PDMAEA-pDNA and compared with PEI-pDNA. It should be noted that the optimized time interval for cell transfection is less than approximately 10 h after complex formation. This is due to the fact that dissociation of polymer and release of pDNA inside the cells usually occurs after 10 h^9^. As shown in Fig. 6, the maximum transfection was observed for an N/P ratio of 50. It has been noted that the rate of transfection with nano-polyplexes is higher than that of complexes with all molecular weights. The results suggest that an increase in the molecular weight and the N/P ratio leads to enhancement of the transfection efficiency. It is noteworthy that higher N/P ratios (100 and 200) with polymers of lower molecular weight induced better transfection efficiency, whereas polymers of higher molecular weights did not show the same efficiency. Findings of this effort are consistent with previous research that reported the use of PDMAEA as well as other polymers such as chitosan, PDMAEMA, and PEI as a gene carrier^3,9,27,28^.

### Dark-Filed imaging and Confocal microscopy

Dark-field microscopy can be utilized to monitor cellular uptake and transfection process by orange-red plasmonic scattering phenomenon that relies on the strong longitudinal surface plasmon resonance of GNRs in the near IR spectrum. Such a characteristic phenomenon enables potential application of GNRs as contrast agents in tracking nanopolyplex systems^29,30^. Figure 6(b) shows dark-field microscopy images of cellular uptake of nano-polyplex system (pDNA-PDMAEA-GNRs) by HEK-293. Cell internalization was also monitored by confocal microscopy as a powerful technique for high resolution optical sectioning of samples and three dimensional detection^31^. This technique provides useful information on location of nanoparticles within cell environment^32^. Figure 6(c) shows confocal microscopy images of HEK-293 cells treated with SYBR Green-labeled nano-polyplex and DAPI stained nuclei, confirming internalization and accumulation of the nano-polyplex around the nuclei.

**Figure 7:**
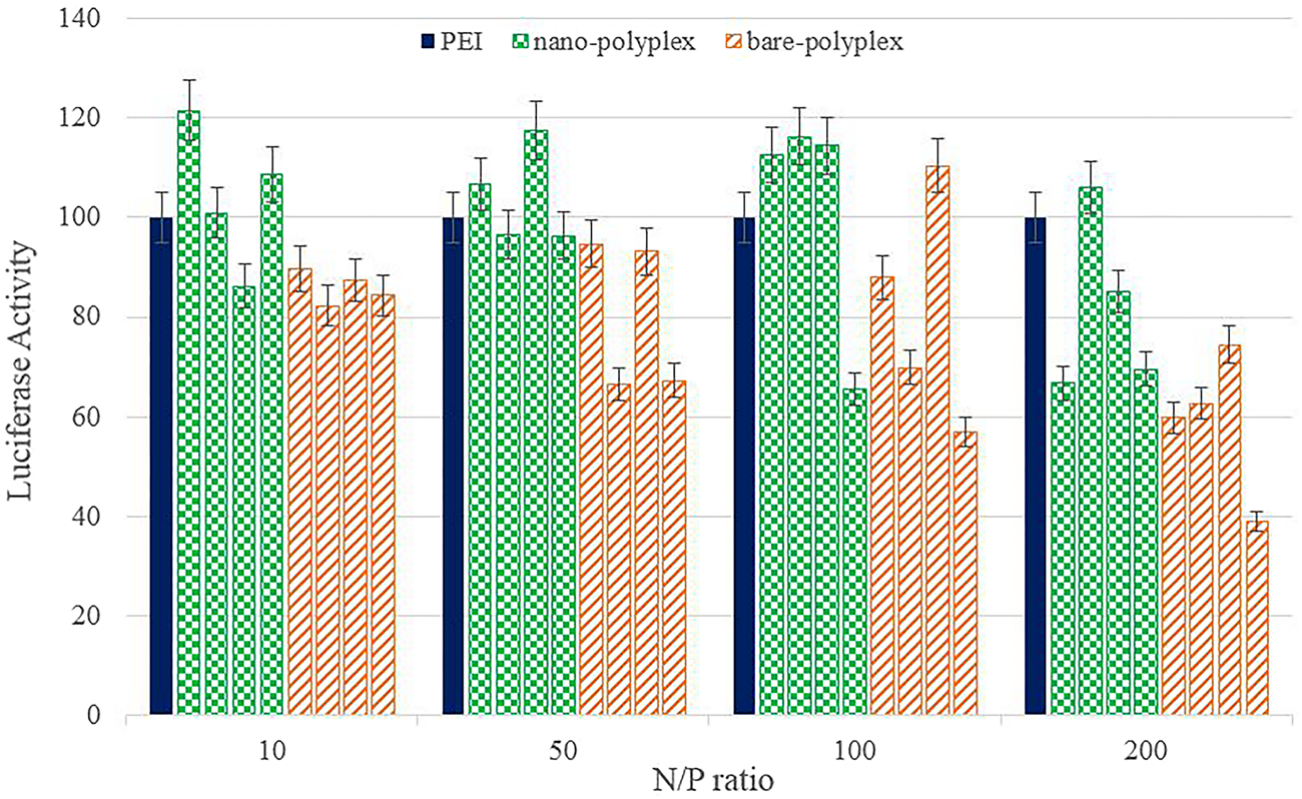
Measurement of transfection efficiency of nano-polyplex, bare-polyplex and pDNA-PEI using luciferase assay in HEK293 cell’s 48 hours post transfection. Different colors indicates PIE (), nano-polyplex (), bare-polyplex () as carriers of pGL3. Four different molecular weights along with specified N/P ratio of PDMAEA were treated with the HEK293 cell line in presence (nano-polyplex) and absence (bare-polyplex) of GNRs. Complexes of pDNA-PEI was used as positive control. As shown in histogram, nano-polyplex with N/P ratio of 50 and 100 presented a better pattern of transfection.

Another evaluation based on reported gene technology includes bioluminescence and luciferase assay which is widely used for gene expression analysis in mammalian cells. Rapid, sensitive and nonradioactive detection indicates that the firefly luciferase gene is a suitable candidate for monitoring promoter activity and observing the activity of living cells^33^. Plasmids of pGL3 were mixed with GNRs-PDMAEA with N/P ratios of 10–200, and incubated with HEK293 cells as described for the GFP gene. Transfection results indicated the effectiveness of the nano-polyplex system in delivery of pGL3 to the HEK293 cell nucleus, and subsequent expression of the luciferase gene (Fig. 7). According to these results, an inverse relationship between molecular weight and N/P ratio is observed, such that increasing the molecular weight is associated with decreasing the proper N/P ratio of transfection. Such behavior is considerable for molecular weights of II, III and IV polymers and N/P ratios of 100, 50 and 10, respectively. However, the overall data represents an important point, i.e. the average transfection of the nano-polyplex system is more than PEI and the bare-polyplex of PDMAEA.

Among nucleic acids, DNA-based therapy offers a better opportunity for gene delivery purposes because of its multivalent treatments^34^. Hence, stability of pDNA during interaction with cell membrane, uptake, intracellular movements and access to nucleus should be considered as a crucial issue^35^. In this regard, the design and fabrication of proper carriers would be the prerequisite for efficient transfection, i.e. the majority of pDNA can be delivered to the target area without any damage. Stability of the carriers during the long extra- or intra-cellular paths is a key factor that defines their fate and performance in biological environments^36^. Different release rates of nucleic acids from cationic nano-carriers are distinctive features of polymers in gene delivery. It has been shown that electrostatic interaction plays an important role in polyplex formation and dissociation in cellular conditions^1^. For instance, PEI with high positive charge can strongly interact with negatively charged nucleic acids with high transfection efficiency among other cationic counterparts. Nonetheless, PEI is found to be highly toxic, creating a great challenge to overcome for fruitful gene delivery purposes^37^. Formation of cavity structures in plasma membranes, membrane ablation and thinning of lipid bilayers are considered common phenomena when cationic carriers interact with anionic phospholipids of cell membranes^38,39^. Protonated polymers such as PEI, PDMAEMA and PAMAM, due to their buffering capacities, show easy endosomal escape. In fact, a phenomenon known as the proton sponge effect is related to the buffering effects of polymers in the acidic environment of endosomes. Subsequently, osmotic swelling and disruption of endosomes lead to release of polyplexes into the cytoplasm and resolve intracellular trafficking^40,41^. Nevertheless, the non-biodegradable nature and toxic effect can be considered as important limiting factors of such carriers systems with considerable proteins inactivation, strong linkage with cellular nucleic acids, and aggregation in cell organelles^42^. Meanwhile, PDMAEA can be regarded as a biocompatible and biodegradable polymer that is a promising carrier candidate in conjunction with GNRs that allows reduced intracellular trafficking and toxicity. Furthermore, the nano-polyplex of GNR-PDMAEA-pDNA simultaneously exploits gold atoms as an inert platform from the biological and chemical points of view^43^ and PDMAEA with features of biocompatibility and self-catalyzed degradability. Another superior trait of the current nano-polyplex system with respect to other reported carriers is its size, which is estimated to be about 100 nm at all N/P ratios of PDMAEA. Previous studies have shown that an optimal size range exist for carriers to improve their cellular uptake. It has also been demonstrated that increasing size of the carrier leads to a decrease in the opportunity for cell entry. Moreover, nanostructures smaller than 100 nm are found to arrive in the cell cytoplasm faster and more easily^44^. Results of this effort are consistent with the above-mentioned studies. Hence, the present nano-polyplex can hopefully pave the way for further investigations on design and fabrication of nanoparticle-based gene delivery systems in the future.

## Materials and Methods

### Materials

Chloroauric acid (HAuCl_4_), sodium borohydride (NaBH_4_), ascorbic acid, hexadecyltrimethylammonium bromide (CTAB), and silver nitrate (AgNO_3_) were purchased from Sigma. Methyl 2 (dodecylthiocarbonothioylthio)-2-methylpropionate 97% (HPLC), 2-(Dimethylamino) ethyl acrylate (98%, Sigma-Aldrich), azobisisobutyronitrile (AIBN), and branched polyethylenimine (25 kDa) were purchased from Sigma. 2-(Dimethylamino) ethyl acrylate was passed through a basic alumina column (activity I) to remove the inhibitor. Azobisisobutyronitrile (AIBN) was recrystallized twice from methanol prior to use. Dioxane (99%) and n-Hexane (>98%) were purchased from Merck. Plasmid DNA (pEGFP-N1 and pGL4) were procured from Invitrogen. All other chemicals and solvents were of analytical grade. Deionized water was used in all the experiments.

### Preparation of gold nanorods (GNRs)

Sequential seed mediated growth methods were used for gold nanorods^45^. In brief, small spherical gold nanoparticles (seeds) were synthesized by mixing aqueous solutions of CTAB (7.5 mL, 0.095 M), HAuCl_4_ (250 μL, 0.01 M) and ice-cold NaBH4 solution (600 μL, 0.01 M), respectively. Two minutes of rapid inversion was necessary for mixing. The reactants were kept undisturbed at room temperature for a minimum of 2 h. The growth solution was prepared by sequential addition of CTAB (9.5 mL, 0.095 M), HAuCl_4_ (400 μL, 0.01 M), AgNO_3_ (60 μL, 0.01 M), ascorbic acid (64 μL 0.10 M), and the seed particles (40 μL). Formation of rod-shaped nanostructures was initiated, accompanied by change in the solution color. It is worth noting that termination of the reaction might take several hours^45^. The average size of GNRs and their distribution was determined by transmission electron microscopy (TEM). The images were analyzed by Image J software.

### Synthesis of Poly (2-(dimethylamino) ethyl acrylate) (PDMAEA)

All four PDMAEAs with different molecular weights (Table 1) were synthesized using the RAFT method, where methyl-2-(dodecylthiocarbonothioylthio)-2-methylpropionate was used as a RAFT agent. To obtain different molecular weights of PDMAEA, the variable parameters were reaction time and molar ratio of RAFT agent to the monomer. For a typical reaction, certain amounts of monomer, RAFT agent and AIBN were dissolved in dioxane as a solvent in a 10 mL Falcon® tube with a magnetic stirrer. The molar ratio of monomer ([DMAEA] 0) to RAFT agent ([RAFT] 0) to AIBN ([AIBN]) was chosen to be [PDMAEA] 0: [RAFT] 0: [AIBN] 0 = 1000:10:1. The reaction mixture was then de-oxygenated by purging with nitrogen for 30 min followed by heating to 60 °C for 7 h. The reaction was stopped by incubation on ice and exposure to air. The precipitating and re-dissolving procedure was repeated three times in n-hexane and dioxane as nonsolvent and solvent, respectively. The polymer was dried under vacuum at 60 °C for 48 h and finally a yellow oily product was obtained. The polymer was characterized by nuclear magnetic resonance spectroscopy (NMR) and gel permeation chromatography (GPC). NMR spectra were recorded by a Bruker Avarce DRX 500 MHz spectrometer, using deuterated chloroform (CDCl_3_) and water (D_2_O) as the solvent for NMR analysis. To analyze the molecular weight distribution of the polymer GPC, tetrahydrofuran (THF) was applied as an eluent. GPC columns were calibrated with narrow molecular weight distribution polystyrene standards in the molecular weight range of polymers.

### Conjugation of Polymer with GNRs

Briefly, 3 mg of polymer were dissolved in 5 ml GNRs (1 OD) and stirred at 200 rpm for 6 hours. The solution was then centrifuged at 13000 rpm for 10 min at room temperature. The precipitate was re-dispersed in 5 ml deionized water. The purification procedure was repeated twice.

### Binding Assay

Plasmids of GFP and luciferase (1 µg) were used to generate polyplexes with PDMAEAs at different N/P ratios (referring to the number of nitrogen (PDMAEA) per DNA phosphate (pDNA) in generated polyplexes). For this purpose, pDNA was complexed with polymers of 10 to 200 N/P ratios, followed by addition of water to the mixture up to 50 μL, and keeping at room temperature for 30 min (without stirring). All polyplexes were loaded onto a 2% agarose gel containing 1X TBE buffer. The gel was run for 1 hour at 80 V, stained with ethidium bromide and visualized using the UV Gel doc (Vilber Lourmat Gel doc, France).

### Protection Assay

Polyplexes were prepared by addition of pDNA (2 μg) to different N/P ratios of polymers with a total reaction of 100 μl, followed by keeping the mixtures at room temperature for 30 min. DNase (1U, Fermentas, Germany), 10X DNase buffer (15 μl) and water (34 μl) were added to each sample and incubated at 37 °C for 10 min. To isolate pDNA, equal volumes of a phenol/chloroform mixture (200 μL of a 1/1 ratio) were added to the polyplexes. Subsequently, the reactions were vortexed (30 seconds) and centrifuged at 12000 rpm at 4 °C for 10 minutes. The supernatant (5 μL) was mixed with DNA loading dye (2 μL), and was loaded onto 2% agarose gel containing 1X TBE buffer. The gel was run in 1X TBE buffer for 1 h at 80 V, as previously mentioned.

### Cytotoxicity Assay

HEK 293 cells (1 × 10^4^) were seeded into 96-well plates for 24 h. The complexes (PDMAEA-pDNA-GNRs and PDMAEA-pDNA) with different N/P ratios (10 to 200) were prepared by addition of pDNA (1 μg). The volume was adjusted to 150 ml with fresh serum-free F12-DMEM. Samples were incubated at room temperature for 30 min. HEK 293 cells were treated with the complexes and incubated for 24 h under optimum growth condition. MTT solution (20 ml, 5 mg/ml) was added to each well and incubated at 37 °C for 4 h. Data was recorded using a micro plate reader (ELx800, Biotek, USA) at 570 nm. PEI was used as the positive control.

### Green Fluorescent Protein Transfection

HEK 293 cells (4 × 10^4^) were seeded into 24 well plates for 48 h before transfection. Complexes were prepared by mixing pDNA (1 μg) with appropriate amounts of GNR- PDMAEAs and PDMAEAs with different N/P ratios (10 to 200), pipetted 10 times and incubated at room temperature for 15 min, this was followed by addition of fresh serum-free F12-DMEM medium to make up a total volume of 100 μl (incubated for 15 more minutes). The complexes were then added to 24-well plates and incubated for 4 h under optimum growth condition (Memmert, Germany). Then 300 μl of F12-DMEM containing serum (Gibco) was added to each well and incubated for 48 h under optimum growth condition. Samples were observed with a fluorescence inverted microscope (Nikon Eclipse TE2000-S fluorescence microscope, Japan).

### Dark-Filed imaging and Confocal microscopy

Briefly, HEK-293 cells were seeded on square glass placed at the bottom of six well plate at 30–40% confluency in complete DMEM-F12 medium. HEK-293 cells were then treated by nano-polyplex (pDNA-PDMAEA-GNRs). After 4 hours of incubation, the transfected cells were fixed with 10% formalin (Mojallali, Iran) and imaged by dark-field microscopy (Olympus, BX51TF, Japan). Likewise, for confocal microscopy the confluent cells were treated by SYBER Green-labeled nano-polyplex. Followed by 4 hours of incubation and fixing with 10% formalin, cells were stained with DAPI (Santa Cruz, sc-24941) for 5 minutes. The images were taken using a spectral confocal microscope (Leica TCS SPE, Germany).

### Assay of Luciferase Activity

The procedure for luciferase activity protein was almost the same as mentioned for GFP. All the experiments including cells seeding, preparation of complexes and cell treatment with complexes were similar. Luciferase plasmid (pGL4) was used and the assay was carried out according to the manufacturer’s instructions (Promega, U.S.A.). Luciferase activity was measured with a luminometer (Berthold Detection Systems, GmbH).

## Conclusion

The rate and efficiency of transfection is an important issue for gene therapy applications. Nano-polyplexes consisting of gold nanorods and PDMAEA were synthesized to monitor the transfection efficiency in the HEK-293T cell line. Results showed that the nano-polyplex presented excellent features with self-catalyzed degradation of PDMAEA in an aqueous medium. The nano-polyplex exploited the efficient binding, protecting and pDNA releasing potential of PDMAEA as well as the prominent features of GNRs, i.e., high surface-area-to-volume ratio and facile functionalizability. The entrance rate of the nano-polyplex increased upon maximized loading of polymers onto GNRs’ matrix, leading to more transfection of pDNA. Comparison of the nano-polyplex system with previously reported data on the bare polyplex of PDMAEA also showed higher level of transfection with respect to the latter. Furthermore, toxicity of the nano-polyplex was found to be lower than that of bare PDMAEA. Results of this effort could pave way for further investigations on the design and fabrication of a new generation of nanoparticle-based delivery systems for gene therapy and gene editing applications.
